# *Aqp9* Gene Deletion Enhances Retinal Ganglion Cell (RGC) Death and Dysfunction Induced by Optic Nerve Crush: Evidence that Aquaporin 9 Acts as an Astrocyte-to-Neuron Lactate Shuttle in Concert with Monocarboxylate Transporters To Support RGC Function and Survival

**DOI:** 10.1007/s12035-020-02030-0

**Published:** 2020-08-04

**Authors:** Sotaro Mori, Takuji Kurimoto, Akiko Miki, Hidetaka Maeda, Sentaro Kusuhara, Makoto Nakamura

**Affiliations:** 1 Division of Ophthalmology, Department of Surgery, Kobe University Graduate School of Medicine, 7-5-1 Kusunoki-cho, Chuo-ku, Kobe, 650-0017 Japan; 2 Maeda Eye Clinic, 1-1-1, Uchihonmachi, Chuo-ku, Osaka, 540-0012 Japan

**Keywords:** Aquaporin 9, Monocarboxylate transporter, Astrocyte-to-neuron lactate shuttle, Optic nerve crush, Retinal ganglion cells, Scotopic threshold response

## Abstract

Aquaporin 9 (AQP9) is an aquaglyceroporin that can transport lactate. Accumulating evidence suggests that astrocyte-to-neuron lactate shuttle (ANLS) plays a critical role in energy metabolism in neurons, including retinal ganglion cells (RGCs). To test the hypothesis that AQP9, in concert with monocarboxylate transporters (MCTs), participates in ANLS to maintain function and survival of RGCs, *Aqp9*-null mice and wild-type (WT) littermates were subjected to optic nerve crush (ONC) with or without intravitreal injection of an MCT2 inhibitor. RGC density was similar between the *Aqp9*-null mice and WT mice without ONC, while ONC resulted in significantly more RGC density reduction in the *Aqp9*-null mice than in the WT mice at day 7. Positive scotopic threshold response (pSTR) amplitude values were similar between the two groups without ONC, but were significantly more reduced in the *Aqp9*-null mice than in the WT mice 7days after ONC. MCT2 inhibitor injection accelerated RGC death and pSTR amplitude reduction only in the WT mice with ONC. Immunolabeling revealed that both RGCs and astrocytes expressed AQP9, that ONC predominantly reduced astrocytic AQP9 expression, and that MCTs 1, 2, and 4 were co-localized with AQP9 at the ganglion cell layer. These retinal MCTs were also co-immunoprecipitated with AQP9 in the WT mice. ONC decreased the co-immunoprecipitation of MCTs 1 and 4, but did not impact co-immunoprecipitation of MCT2. Retinal glucose transporter 1 expression was increased in *Aqp9*-null mice. *Aqp9* gene deletion reduced and increased the intraretinal l-lactate and d-glucose concentrations, respectively. Results suggest that AQP9 acts as the ANLS to maintain function and survival of RGCs.

## Introduction

Accumulating evidence indicates that some neurons in the central nervous system prefer lactate over glucose as an energy substrate and that astrocyte-to-neuron lactate shuttle (ANLS) plays a critical role in maintaining neuronal metabolism, function, and survival [1, 2]. In ANLS, astrocytes are thought to take up glucose from blood vessels, convert it to lactate, and release the lactate to neurons. The neurons then convert the lactate into pyruvate, which can enter the tricarboxylic acid (TCA) cycle to produce adenosine triphosphate (ATP) [1, 2]. Although the ANLS hypothesis is not fully accepted as the mode of energy metabolism in the outer retina, particularly in photoreceptors [3], the role of ANLS in the inner retina, and particularly in retinal ganglion cells (RGCs), has been increasingly a matter of great interest, given the intimate association of astrocytes and the unmyelinated axons of RGCs in the inner retina.

Aquaporin 9 (AQP9) is one of 13 members in the AQP family of water channels. However, it belongs to an aquaglyceroporin, which is permeable not only to water but also to monocarboxylates including glycerol and lactate, and ketone bodies [4, 5]. AQP9 is reported to be expressed in astrocyte cell bodies and astrocytic processes of the brain white matter, including the optic chiasm [5–8], as well as in cell bodies and dendrites of the substantia nigra and dopaminergic neurons [9]. Catecholaminergic amacrine cells were the first reported cells in the retina to express AQP9 [10]). Subsequent studies by our group [11, 12] and others [13] identified that RGCs also express AQP9. In addition, it has been reported that elevated intraocular pressure (IOP) and optic nerve transection in rodents [11, 12], and glaucoma in humans [13], lead to reduced AQP9 expression in RGCs. Further, we previously demonstrated that AQP9 expression is required for l-lactate to maintain survival of RGCs in culture [14]. These lines of evidence implicate AQP9 as a lactate transporter in ANLS in the inner retina.

Key players in lactate transport and ANLS are thought to be members of a family of proton-linked monocarboxylate transporters (MCTs) [15, 16]. In the brain, it is known that endothelial cells and glia express MCT1 and MCT4, whereas neurons express MCT2 [17]. MCTs 1 and 4 are presumed to primarily release lactate. In contrast, MCT2 takes up lactate [17, 18]. Previous studies demonstrated that in the retina, MCT1 is expressed by retinal pigment epithelium (RPE), inner segments of photoreceptors, Müller cells, and the inner and outer plexiform layers (IPL and OPL), while MCT2 is expressed in the endfeet of Müller cells, astrocytic processes surrounding endothelial cells [19–21], and MCT4 is expressed in the inner retina [22]. It is not known whether or how AQP9 and MCTs interact with each other to facilitate ANLS in the retina.

Glucose, the classical energy substrate for neurons, is transported by glucose transporter (GLUT) family members [23]. Among the subtypes of GLUTs, neurons are reported to specifically express GLUT3, while astrocytes and endothelial cells express GLUT1 [24–26]. However, it is unknown how stresses on the optic nerve affect the expression levels of AQP9, MCTs, and GLUTs and alter glucose and lactate utility in the retina, which ultimately impact survival and function of RGCs.

This study was to use *Aqp9*-null mice and an optic nerve crush (ONC) model to determine whether AQP9, in concert with MCTs, plays a critical role in ANLS and helps to maintain function and survival of RGCs.

## Methods

### Animals

*Aqp9* knockout (KO; *aqp9*^*−/−*^) mice were gifted from Professor Søren Nielsen from the Department of Biomedince of Aarhus University, Denmark. These mice have a C57BL/6J genetic background with targeted gene disruption of *aqp9*, as described by Rojek et al. [27], and exhibit normal development, fertility, appearance, and behavior under physiological conditions. Contents in plasma are within normal range except for an increase in glycerol and triacylglycerol levels [27]. The experiments used 20- to 25-week-old male *Aqp9* KO mice and their wild-type (WT) littermates (*aqp9*^*+/+*^) (body weight, 25–35 g). Mice were housed in the Kobe University Animal Facility with ad libitum access to food and water under a 12-h light/12-h dark cycle at room temperature (24 ± 2 °C). Body weight and blood glucose levels were measured between 9 am and 11 am once per week. IOP was measured in awake mice using a rebound tonometer (TonoLab; Tiolat, Helsinki, Finland), as previously reported [11]. In each session, the tonometer took six measurements using an internal software that eliminated the highest and lowest readings and calculated a mean of the remaining values. After recording five consecutive sessions, the five mean IOPs were averaged, which was defined as the IOP at the specific time point.

Animal experiments were approved by the Animal Care Committee of the Kobe University Graduate School of Medicine and were conducted in accordance with the guidelines set forth in the Association for Research in Vision and Ophthalmology Resolution on Care and Use of Laboratory Animals.

### Optic Nerve Crush

Mice were anesthetized by intraperitoneal injection of ketamine (100 mg/kg) and xylazine (10 mg/kg). Optic nerve surgery was performed as previously reported [28, 29]. In brief, after resecting the superior rectus muscle, the left optic nerve was exposed and crushed with forceps 0.5 mm behind the eyeball for 10 s. Normal retinal circulation after crush was confirmed by dilated ophthalmoscopic examination and later by electroretinogram as described below in detail. At the appropriate period, mice were sacrificed and perfused by 4% paraformaldehyde (PFA). The retinas were carefully dissected for further analysis.

### Intravitreal Injection of Monocarboxylate Inhibitor

α-Cyano-4-hydroxycinnamate (4-CIN; Sigma-Aldrich, St. Louis, MO, USA) is a competitive, non-transportable inhibitor of MCTs that inhibits mitochondrial lactate and pyruvate transport. Previous reports have shown that 4-CIN relatively selectively inhibits MCT2 rather than MCTs 1 and 4 due to the difference in IC_50_ values (the concentration of agent required to reduce lactate transport by 50%) among the MCT isoforms [30–32]. Similar to a previous report [33], 1 μL of 10 mM 4-CIN dissolved in dimethyl sulfoxide (DMSO) or vehicle (DMSO alone) was intravitreally injected into the eyes immediately after ONC, being careful to avoid lens damage. In accordance with the study of Erlichman et al. [30], the IC50 value of 4-CIN was 425 M for MCT1 and 900 mM for MCT4, whereas it was as low as 24 μM for MCT2. Therefore, the current concentration of 10 mM was deemed to adequately impede MCT2 function but has little, if any, effect on the inhibition of MCTs 1 and 4.

### Reverse-Transcription Polymerase Chain Reaction

Total ribonucleic acid (RNA) from whole retinas was extracted using RNeasy (catalog # 74134, Qiagen, Hilden, Germany) and was reverse-transcribed using a QuantiTect reverse transcription kit (catalog # 205310, Qiagen) [11, 12]. PCR was performed using TaqMan universal master mix (catalog # 436910, Thermo Fisher Scientific, Waltham, MA, USA). Aliquots of the amplicons were electrophoresed in 2% agarose gel with ethidium bromide and were visualized under ultraviolet light. The forward primer sequence for *Aqp9* was 5′-TCAGTCGAGAAAAGGCTGGT-3′ and the reverse primer sequence was 5′-GGCACGGATACAATGGTTT-3′. Glyceraldehyde 3-phosphate dehydrogenase (*Gapdh*) was used as an internal control. The thermal cycling conditions consisted of 40 cycles of denaturation at 95 °C for 1 s and annealing and extension at 60 °C for 20 s using a Mastercycler (Eppendorf, Hamburg, Germany). The anticipated lengths of the amplicons were 221 base pairs for *Aqp9* and 87 base pairs for *Gapdh*.

### Immunohistochemistry

Retinal cryosections (8 μm thick) were collected on glass slides and fixed with 4% PFA for 10 min. After blocking with 5% bovine serum albumin (BSA), the sections were incubated at 4 °C overnight with an appropriate concentration of primary antibodies, as listed in Table 1. Following three 10 min washes with phosphate-buffered saline containing 0.1% Triton X-100 (PBS-T), the sections were incubated with secondary antibodies at room temperature for 1 h [11, 12, 34]. Thereafter, the sections were washed for three times with PBS-T, counterstained with 4′,6-diamidino-2-phenylindole (DAPI; Vector Laboratories, Burlingame, CA, USA), and mounted with coverslips.

**Table 1:** List of antibodies used for immunostaining, immunoprecipitation, and western blot

Antibody	Dilution and purpose	Catalog No.	Company	Host
AQP9	1:200, IHC	ab15129	Abcam	Chicken
AQP9	1:40, IP, 1:5000, WB	ab84828	Abcam	Rabbit
Brn3a	1:100, IHC	sc-8429	Santa Cruz	Mouse
β-Actin	1:200, WB	ab115777	Abcam	Rabbit
GFAP	1:400, IHC	C9205	Sigma-Aldrich	Rabbit
GLUT1	1:200, IHC,1:5000, WB	B300-666	Novus Biologicals	Rabbit
GLUT3	1:200, IHC, 1:2000, WB	NBP2-66872	Novus Biologicals	Rabbit
MCT1	1:500, IHC, 1:1000, WB	bs-10249R	Bioss	Rabbit
MCT2	1:200, IHC, 1:1000, WB	bs-3995R	Bioss	Rabbit
MCT4	1:200, IHC, 1:1000, WB	bs-2698R	Bioss	Rabbit
RBPMS	1:500, IHC	GTX118619	GeneTex	Rabbit
TUBB3	1:500, IHC,	488-435L	Biolegend	Mouse
AF488-anti-rabbit IgG	1:500, IHC	A-21206	Thermo Fisher Scientific	Donkey
AF488-anti-chicken IgG	1:500, IHC	A-11039	Thermo Fisher Scientific	Goat
AF594-anti-rabbit IgG	1:500, IHC	AB2534095	Thermo Fisher Scientific	Goat

*AF* Alexa Fluor, *IHC* immunohistochemistry, *IP* immunoprecipitation, *WB* western blotting

### Immunoprecipitation

To investigate whether AQP9 interacts with MCTs, immunoprecipitation (IP) was performed using Dynabeads Protein A Immunoprecipitation Kit (catalog # 10006D, Invitrogen, Carlsbad, CA, USA) according to the manufacturer’s instruction. In brief, the retinas were homogenized by ultrasonication in lysate buffer (0.1 M sucrose, 0.1 M EDTA, 20 mM Tris-HCl pH 7.5, 2.5 mM sodium pyrophosphate, and 1 tablet of Complete tablets EDTA-free protease inhibitor cocktail (catalog # 4693132001; Sigma-Aldrich)). After centrifugation at 1000*g* for 5 min, the collected supernatant was incubated in 1% sodium dodecyl sulfate (SDS) for 30 min. Then, the samples were centrifuged at 23000*g* for 30 min. Protein concentrations of the supernatants were quantified with a Nanodrop Lite (Thermo Fisher Scientific). Retinal lysate samples containing 800 μg total protein were incubated at room temperature for 10 min with 2 μL of anti-AQP9 antibody (catalog # ab84828, Abcam; Table 1), followed by incubation with Dynabeads Protein A for 10 min. After centrifugation, supernatants containing excess unconjugated antibody were removed, while the tube was placed on a magnet to collect the beads at the bottom of the tube. The elution of Dynabeads-antibody-antigen complexes was used for further immunoblot analysis.

### Immunoblot Analysis

Retinal protein (60 μg) from whole-cell homogenates, or the elution of Dynabeads-antibody-antigen complexes (as described above), was incubated in Laemmli loading buffer with 20 mM dithiothreitol (DTT) for 5 min at 95 °C and then separated by sodium dodecyl sulfate-polyacrylamide gel electrophoresis (SDS-PAGE) (catalog # XP08160BOX, Thermo Fisher Scientific) and transferred to polyvinylidene di-fluoride (PVDF) membranes (catalog # 10600123, GE Healthcare Life Sciences, Buckinghamshire, UK) [12, 35]. The membranes were blocked with 5% BSA in Tris-buffered saline containing 0.1% Tween 20 (TBST) at room temperature for 1 h and then incubated overnight at 4 °C with a primary antibody (against AQP9; MCTs1, 2, and 4; GLUTs1 and 3; and β-actin, as listed in Table 1) in TBST. After three washes with TBST, each for 10 min, the membranes were incubated with horseradish peroxidase-conjugated anti-rabbit IgG (1:2000) for 1 h at room temperature. After four 15-min washes, chemiluminescence detection was performed using ECL reagents (catalog # RPN2232, GE Healthcare Life Sciences) and the signals were quantified using β-actin expression as a reference [12, 35]. Signals were obtained and analyzed using a LAS-3000 Mini digital imaging system (FujiFilm, Tokyo, Japan).

### RGC Labeling

To visualize RGCs, immunolabeling with anti-tubulin β3 (TUBB3) antibody (Biolegend, San Diego, CA, USA) and retrograde labeling with FluoroGold (FG; catalog # 52-9400; FLUOROCHROME, Inc., Denver, CO, USA) were performed. After dissection and blocking in PBS-T with 5% BSA, the flat-mounted retinas were incubated with Alexa Fluor 488-conjugated anti-TUBB3 antibody (1:500) in PBS-T at 4 °C overnight, extensively washed, and then mounted on glass slides.

Retrograde labeling of RGCs was performed with the injection of FG into the superior colliculus (SC) 7 days before ONC, as previously described [36]. In brief, anesthetized mice were placed in a sterotactic apparatus (Narushige Co., Tokyo, Japan). The skull skin was incised, and 2 μL of 2% FG was injected through the exposed brain surface bilaterally into the SC. At 7 days after ONC, retinas were dissected, fixed with 4% PFA, and washed with PBS. The retinas were then extended on a glass slide with the ganglion cell layer (GCL) facing up and mounted.

The images of TUBB3-positive and FG-positive cells at eight pre-specified flat-mounted retinal areas were taken under a fluorescence microscope (BioZero, Keyence, Osaka, Japan) as follows. An observer (T.K.), masked to the treatment conditions of the mice, counted TUBB3- or FG-positive cells at two points (1 mm and 2 mm from the optic disc) in each quadrant (temporal, nasal, inferior, and superior) retinal area using a 40× objective lens, i.e., 0.145 mm^2^ per visual field and a total of eight visual fields per retina. Based on these images, the averaged cell densities were calculated using the NIH ImageJ software (National Institutes of Health, Bethesda, MD). The coefficient of error [37] was less than 0.05, ensuring that our sampling rate was sufficient.

### Electroretinogram Recordings

Mice were dark-adapted overnight before testing and all procedures were performed under dim red light, as previously reported, with a modification [38]. In brief, on the day of experiment, mice were anesthetized with intraperitoneal injection of ketamine (100 mg/kg) and xylazine (10 mg/kg) and positioned on the recording apparatus (Celeris, Diagnosys LLC, Gaithersburg, MD, USA) [39]. Pupils were dilated using eye drops of 0.5% tropicamide and 0.5% phenylephrine hydrochloride (Santen Pharmaceutical Co., Osaka, Japan) and corneas protected by application of a thin layer of methylcellulose. Body temperature was constantly maintained at 37 °C with a built-in heating pad. Full-field illumination of the bilateral eyes was achieved with miniaturized Ganzfeld spheres integrated with the recording electrodes (Celeris Bright RGB stimulators, Diagnosys LLC). ERG responses were analyzed with the Espion software (Diagnosys). Responses were amplified differentially, band-pass filtered with 0.125–50 Hz for STR recordings and 0.125–300 Hz for higher intensity stimulations of dark-adapted response recordings, digitized at 10 kHz, and stored on disk for further processing. Responses to flashes were averaged with an interstimulus interval ranging from 1 s for dim light to 10 s for the brightest flashes. For recording STR, serially increasing luminescence intensities of − 6.1, − 5.5, − 5.1, − 4.6, and − 4.1 log sc td s were used and responses from 80 repeated stimuli for each intensity were averaged. For recording dark-adapted responses, we used luminescence intensities of − 1.15 and − 0.15 log sc td s, and responses from three repeated stimuli at each light intensity were recorded and averaged.

### Colorimetric Assays for d-Glucose and l-Lactate Levels in the Retina

Colorimetric assays to measure d-glucose and l-lactate concentrations were performed using a Glucose Assay Kit (catalog # EBGL-100, Bio-Assay Systems, Hayward, CA, USA) and l-Lactate Assay Kit (catalog # 700510, Cayman Chemical, Ann Arbor, MI, USA), respectively, according to the manufacturer’s instructions. In brief, 100 μg of retinal protein extracts were used. For the glucose assays, retinal lysates, 85 μL of assay buffer, 1 μL of enzyme mix, and 1 μL of the dye reagent were mixed and incubated for 30 min at room temperature. For the lactate assays, retinal lysates were incubated with a solution containing the fluorometric substrate conjugated with lactate dehydrogenase for 20 min. These materials were measured using a spectral photometer (MTP-800; Corona Electric, Ibaraki, Japan; fluorescence intensity *λ*_em/ex_ = 585/530 nm). d-Glucose and l-lactate concentrations were extrapolated from a standard curve that was drawn by the 3 or 4 measured values of known concentrations of these molecules, respectively. The ratios of d-glucose and l-lactate relative to samples from controls (i.e., WT mice that underwent the sham operation with the vehicle injection) were calculated.

### Statistical Analyses

Data are reported as means ± standard error of the mean. Statistical analyses were performed using Excel software version 2013 and MedCalc software version 19.0.3 (MedCalc Software, Ostend, Belgium). Statistical comparisons were made with one- or two-way analysis of variance (ANOVA) when three or more groups were compared and with unpaired *t* tests when two groups were compared. The Bonferroni test was used as for post hoc analysis. A *P* value of < 0.05 was judged to be statistically significant.

## Results

### Confirmation of Retinal AQP9 Expression in WT Mice and Its Absence in *Aqp9* KO Mice

**Fig. 1:**
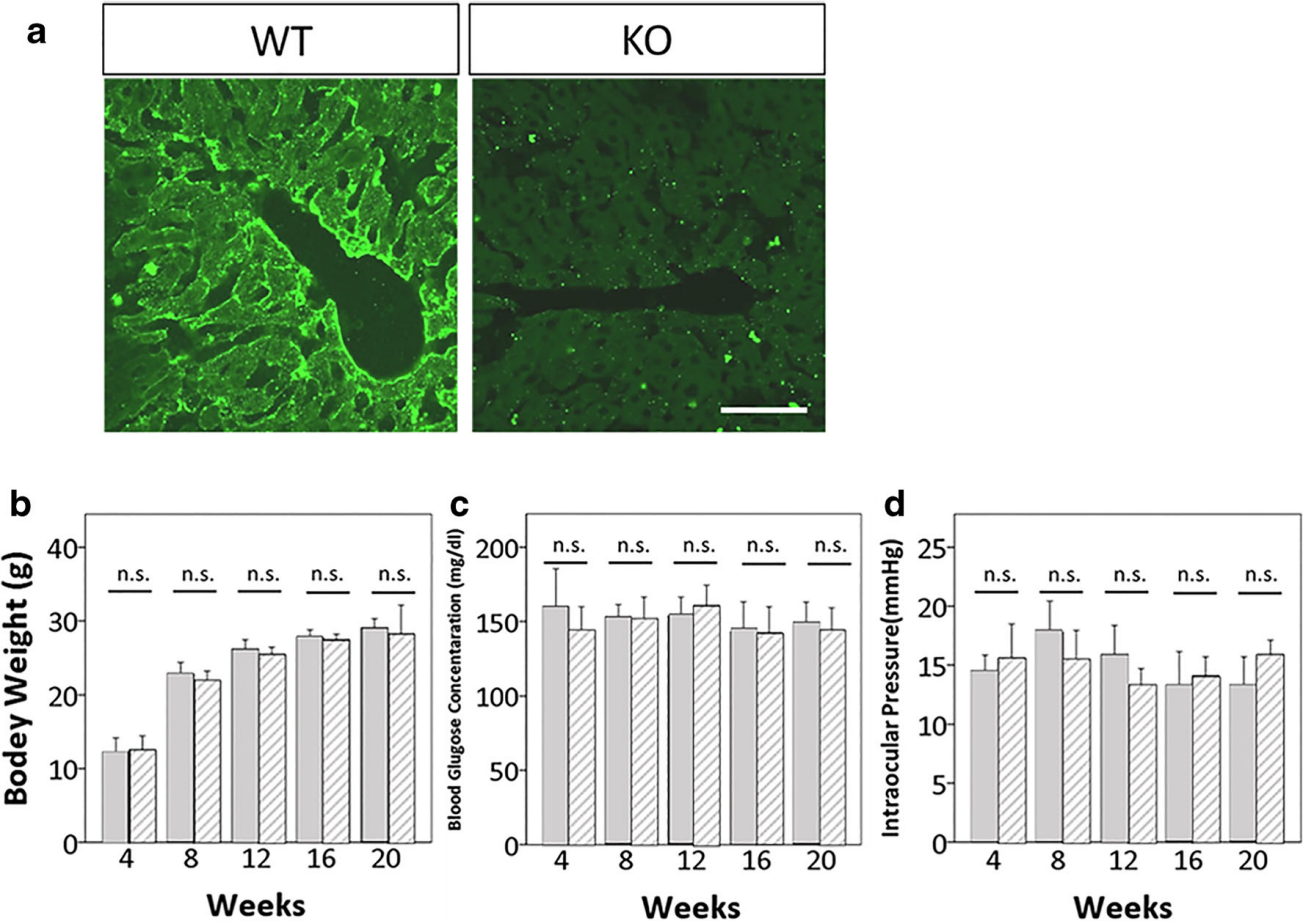
Physiological condition of *Aqp9* KO mice. **a** Immunostaining of AQP9 in liver. WT mice show intense AQP9 immunoreactivity (IR), whereas in *Aqp9* KO mice, IR is almost completely lost. Scale bar indicates 50 μm. **b**–**d** Time course of changes of parameters in WT and *Aqp9* KO mice. **b** Body weight. **c** Blood glucose concentrations. **d**. Intraocular pressures. Blood glucose levels and intraocular pressures were measured in awake mice between 9 am and 11 am. Error bars indicate standard error of the mean (*n* = 10 each). N.S., not significant

We confirmed the positive and null immunoreactivity of AQP9 in liver of WT and *Aqp9* KO mice, respectively (Fig. 1a), which agrees with a previous report [27]. There were no significant differences in IOP, body weight, or blood glucose concentrations between WT and *Aqp9* KO mice at any stage of the experiment (Fig. 1b–d). These findings corroborate previous observations that *Aqp9* KO mice exhibit normal development, appearance, metabolism, and behavior under physiological conditions [27].

**Fig. 2:**
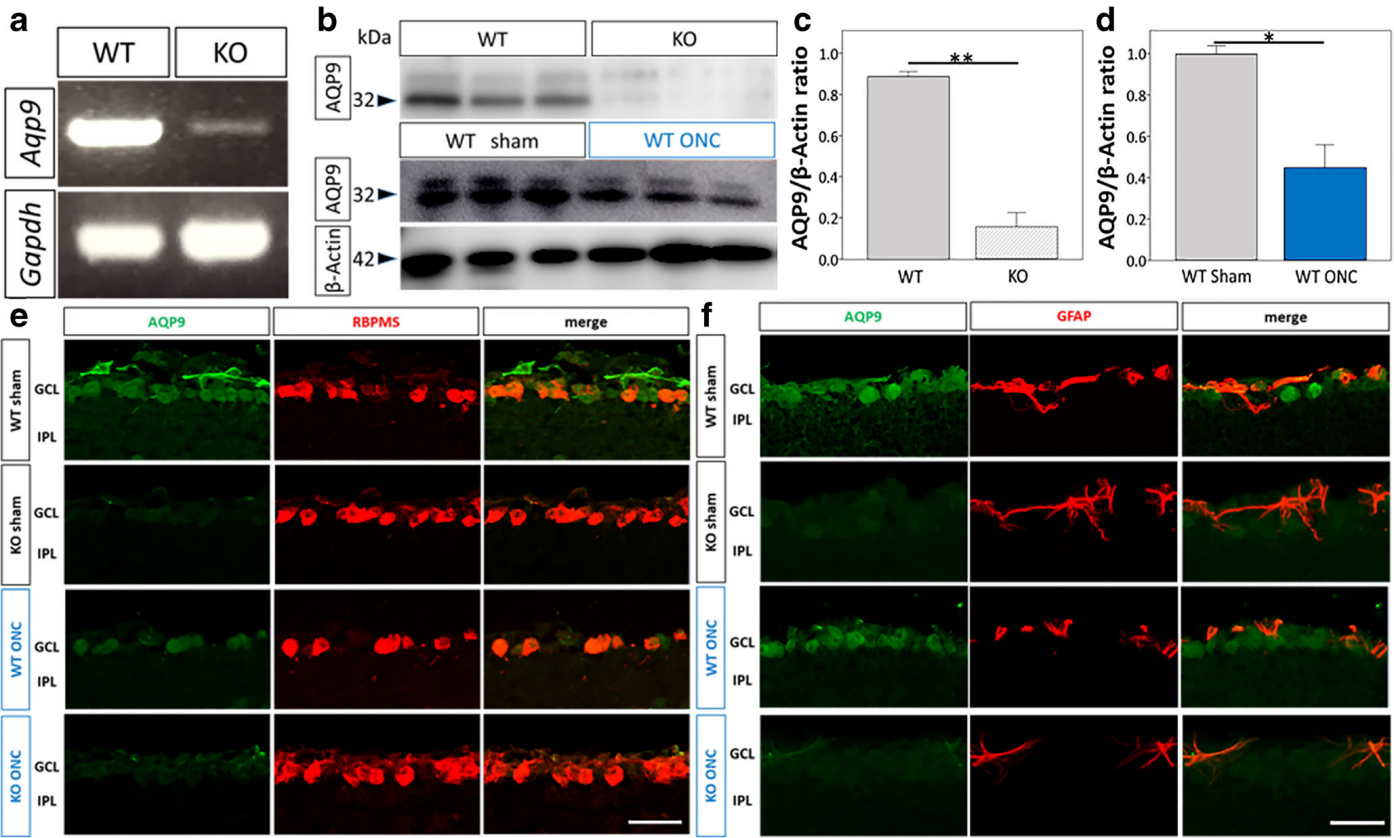
Effect of *Aqp9* gene deletion and ONC on AQP9 expression in retina. **a** RT-PCR and 2% agarose gel electrophoresis to confirm *Aqp9* gene deletion in *Aqp9* KO mice (KO). *Gapdh* was used as a loading control. **b** Western blot analysis. The upper panel shows constitutive and decreased expression of AQP9 protein in retinal homogenates of WT and *Aqp9* KO mice, respectively. The middle panel compares retinal AQP9 expression between WT mice with sham operation (WT sham) and those that underwent ONC (WT ONC) 1 week prior. The bottom panel depicts β-actin protein expression, which was used as an internal control. **c** Quantitative comparison of an AQP9/β-actin ratio in the western blotting data between WT (gray solid bar) and *Aqp9* KO (hatched bar) mouse retinas. Error bars designate standard error of the mean (SEM). *n* = 3 each, **p < 0.0001, unpaired *t* test. **d** Quantitative comparison of an AQP9/β-actin ratio in the western blotting data between WT mice with sham operation (sham: gray bar) and those that underwent ONC (ONC: blue bar) 1 week prior. Error bars designate SEM. *n* = 3 each, *p < 0.01, unpaired *t* test. **e** AQP9 and RBPMS immunoreactivity (IR) in inner retinas. **f** AQP9 and GFAP IR in the inner retinas. Scale bars indicate 25 μm

To verify retinal expression of AQP9 in WT mice and its deletion in *Aqp9* KO mice, we performed RT-PCR and immunoblotting, which revealed that the WT mice had abundant retinal expression of AQP9 both at the gene and protein levels (Fig. 2a, b). In contrast, retinal mRNA and protein expression levels of AQP9 were substantially reduced and almost completely extinguished, respectively, in the *Aqp9* KO mice (*n* = 3, *p* < 0.0001, unpaired *t* test; Fig. 2a–c).

Next, we examined whether or not ONC altered AQP9 protein expression in the retinas of WT mice. Retinas obtained 7 days after ONC were subjected to immunoblotting, which demonstrated that ONC decreased AQP9 protein expression by approximately 40% (*n* = 3, *p* < 0.01, unpaired *t* test; Fig. 2b, d). To identify cell types that express AQP9 in the retina and examine the effects of *Aqp9* deletion and/or ONC on its expression, co-immunolabeling of AQP9 with RNA-binding protein with multiple splicing (RBPMS), an RGC-specific marker, or glial fibrillary acidic protein (GFAP), an astrocyte marker, was conducted (Fig. 2e, f). AQP9 immunoreactivity (IR) was observed in GCL in WT mouse retinas. Nearly all RBPMS- and GFAP-immunoreactive cells exhibited AQP9 IR in the WT mouse retinas without ONC. AQP9 IR was barely detected in *Aqp9* KO mouse retinas, irrespective of ONC or sham operation, although RBPMS- and GFAP-immunoreactive cells were still present. Co-immunolabeling of AQP9 with RBPMS was still observed, whereas the co-immunolabeling of AQP9 with GFAP was prominently reduced in WT mice with ONC, indicating that ONC preferentially diminished astrocytic expression of AQP9 over RGC expression of AQP9.

### AQP9 Is at Least Partly Co-expressed with MCTs1, 2, and 4 in the Retina and *Aqp9* Gene Deletion or/and ONC Affect Their Association in an MCT Isoform-Dependent Fashion

Next, we examined whether MCTs 1, 2, and 4 are expressed, in addition to AQP9, in the inner retina where they could act as additional routes of monocarboxylate delivery to RGCs.

**Fig. 3:**
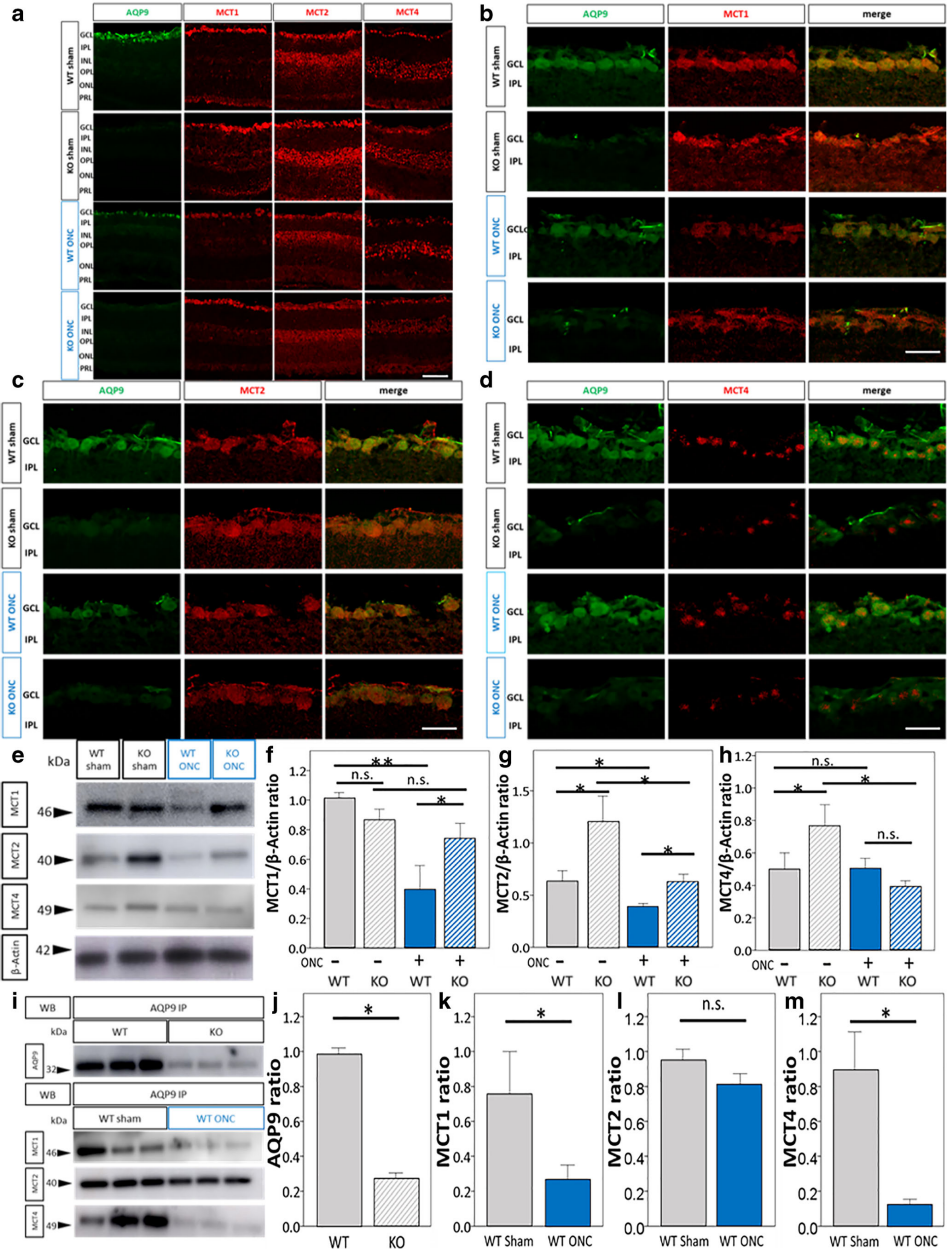
Expression of AQP9 and MCT1, 2, and 4 and effect of *Aqp9* gene deletion and ONC on their expression. **a** Immunoreactivity (IR) of AQP9 and MCTs in WT and *Aqp9* KO mice 7 days after sham operation (WT sham and KO sham, respectively) and ONC (WT ONC and KO ONC, respectively). A scale bar indicates 50 μm. **b**–**d** Co-localization of MCT1, MCT2, and MCT4, respectively, with AQP9 in GCL. Scale bars indicate 25 μm. Note that all isoforms of MCTs are expressed in GCL and at least partly co-labeled with AQP9 in WT mice with the sham operation. **e** Representative western blots for AQP9 and MCTs1, 2, and 4. WT sham, WT mice with a sham operation; KO sham, *Aqp9* KO mice with a sham operation; WT ONC, WT mice 7 days after ONC; KO ONC, *Aqp9* KO mice 7 days after ONC. β-Actin was used as an internal control. **f**–**h** Quantitative analyses for MCT1, MCT2, and MCT4 expression levels, respectively, relative to β-actin levels. Error bars represent standard error of the mean (SEM). *n* = 5; **p < 0.001, *p < 0.01, ANOVA with the Bonferroni post hoc test. **i** Immunoprecipitation (IP) of retinal homogenates with an anti-AQP9 antibody. The upper panel shows auto-probing with the anti-AQP9 antibody for the IPs, while the lower panel depicts western blotting for the IPs with MCT1, MCT2, and MCT4 antibodies. Note that all of the MCTs (1, 2, and 4) are detectable in the IPs for AQP9 in WT sham. **j**–**m** Quantitative analyses for AQP9, MCT1, MCT2, and MCT4 levels, respectively, in the IPs of AQP9 relative to the respective levels in WT mice with sham operation. Error bars indicate SEM. *n* = 3; *p* < 0.001, unpaired *t* test. n.s., not significant

Figure 3a depicts overall IR for AQP9 and MCTs 1, 2, and 4 in the entire retinas in WT and *Aqp9* KO mice with and without ONC. Again, intense AQP9 IR was confined to the GCL in the WT mice without ONC, which was reduced in mice with ONC, as mentioned above. For MCT1 IR, the GCL and photoreceptor layer (PRL) had intense and abundant IR, respectively. IPL and OPL also showed faint MCT1 IR in the WT mice without ONC. These MCT1 expression patterns looked similar between the WT and *Aqp9* KO mice with sham operation. On the other hand, MCT1 IR at the GCL is reduced after ONC, both in the WT and *Aqp9* KO mice. Regarding MCT2, moderate IR was observed in the GCL, the inner nuclear layer (INL), OPL, and PRL in WT mice with the sham operation. ONC did not seem to affect these IR patterns in the WT mice. However, these IR intensities were enhanced and the outer nuclear layer (ONL) also showed MCT2 IR in the *Aqp9* KO mice, both with and without ONC. Regarding MCT4, punctate IR was observed in the GCL, INL, OPL, and PRL in the WT mice with sham operation. These patterns looked similar among the other mouse conditions.

To focus on the association between AQP9 and MCTs, co-immunostaining assays of AQP9 and three isoforms of MCTs were conducted. IR for the three MCT isoforms was at least partly co-localized with AQP9 IR. For MCT1, the WT mice with sham operation seemed to have the most intense IR compared with the remaining three mouse conditions (Fig. 3b). For MCT2 IR, the *Aqp9* KO mice with sham operation seemed to show the highest intensity (Fig. 3c). For MCT4 IR, the intensity did not differ among groups, but the number of MCT4-immunoreactive cells in *Aqp9* KO mice was fewer than that in WT mice, irrespective of ONC or sham operation (Fig. 3d).

Immunoblotting also revealed that the three isoforms of MCTs were expressed in the retinas of WT mice with the sham operation (Fig. 3e–h). Immunoprecipitation with anti-AQP9 antibody followed by immunoblotting for AQP9 or MCTs 1, 2, or 4 confirmed that expression of AQP9 was almost completely extinguished in the *Aqp9* KO mouse retinas (*n* = 3, *p* < 0.001; Fig. 3i, j) and also demonstrated that all three isoforms of the MCTs co-immunoprecipitated with AQP9 (Fig. 3i). These findings show that the MCT proteins at least partly interact with AQP9 in WT mouse retinas under physiological conditions.

Western blotting of whole retinal homogenates demonstrated that MCT1 levels relative to β-actin in the WT mice with ONC were significantly lower than those in the WT mice with the sham operation (*n* = 5, *p* < 0.001, Fig. 3e, f). ONC also reduced MCT1 levels co-immunoprecipitated with AQP9 in WT mice (*n* = 3, *p* < 0.001; Fig. 3i, k), which corresponded to the reduced expression of total MCT1 and AQP9 induced by ONC, as previously mentioned (Fig. 2b, b and Fig. 3a, b). In comparison, the total retinal MCT1 contents in the *Aqp9* KO mice without ONC were similar to those in the WT counterparts despite the lack in AQP9 expression (Fig. 3e, f). In addition, unlike the WT mice, ONC did not reduce the total retinal MCT1 levels in the *Aqp9* KO mice (Fig. 3e, f). These results suggest that MCT1 molecules that do not interact with AQP9 also exist, that their expression levels are upregulated in *Aqp9* KO mice under physiological conditions to possibly compensate for lowered availability of AQP9, and that upregulated MCT1 expression irrelevant to the AQP9 expression is unchanged after ONC in these mice.

The expression patterns of MCT2 were quite different from MCT1 among the different conditions. Total MCT2 levels were significantly reduced in WT mice with ONC compared to those without ONC (*n* = 5, *p* < 0.01; Fig. 3e, g). However, the level of MCT2 co-immunoprecipitated with AQP9 in the WT mice with ONC was not changed compared to WT mice without ONC (Fig. 3i, l). Further, the total retinal MCT2 levels in the *Aqp9* KO mice was significantly higher than those in WT mice with sham operation (*n* = 5, *p* < 0.01; Fig. 3e, g). ONC significantly reduced total MCT2 levels in *Aqp9* KO mice (*n* = 5, *p* < 0.01; Fig. 3e, g), but the expression levels were still significantly higher than in the WT counterparts (*n* = 5, *p* < 0.01; Fig. 3e, g). These results suggest that MCT2 has a high affinity for AQP9 and that ONC mainly downregulates MCT2 that does not interact with AQP9.

Total retinal levels of MCT4 were unchanged by ONC in the WT mice (Fig. 3e, h). However, MCT4 co-immunoprecipitated with AQP9 was almost gone after ONC in the WT mice (*n* = 3, *p* < 0.001; Fig. 3i, m), suggesting that the MCT4 proteins that do not interact with AQP9 were upregulated in WT mice with ONC. In comparison, the total retinal MCT4 levels in *Aqp9* KO mice was significantly higher than in WT mice with sham operation (*n* = 5, *p* < 0.01; Fig. 3e, h), suggesting that the lowered availability of AQP9 also enhanced expression of MCT4 that does not associated with AQP9. ONC significantly downregulated the total MCT4 expression in the *Aqp9* KO mouse retinas compared with the *Aqp9* KO mice without ONC (*n* = 5, *p* < 0.01; Fig. 3e, h), suggesting that ONC may perturb the compensatory upregulation of MCT4 expression in *Aqp9* KO mice.

Collectively, MCTs that interact with AQP9 and those that are expressed independently from AQP9 are both present in the retina. *Aqp9* gene deletion seemed to enhance expression levels of all the three MCT isoforms that are not associated with AQP9, possibly to compensate for the lowered availability of AQP9 as a lactate transporter. MCT2 seemed to have the highest affinity with AQP9. ONC reduced the association of MCT1 with AQP9, but increased MCT1 expression that is uncoupled with AQP9. ONC did not influence the association of MCT2 with AQP9, but reduced MCT2 expression uncoupled with AQP9. ONC broke down the association of MCT4 with AQP9, but possibly increased the MCT4 expression uncoupled with AQP9 in the WT mice. Such a possible compensatory upregulation of MCT4 did not seem to occur in the *Aqp9* KO mouse retinas.

### *Aqp9* Gene Deletion Enhances RGC Death After ONC

**Fig. 4:**
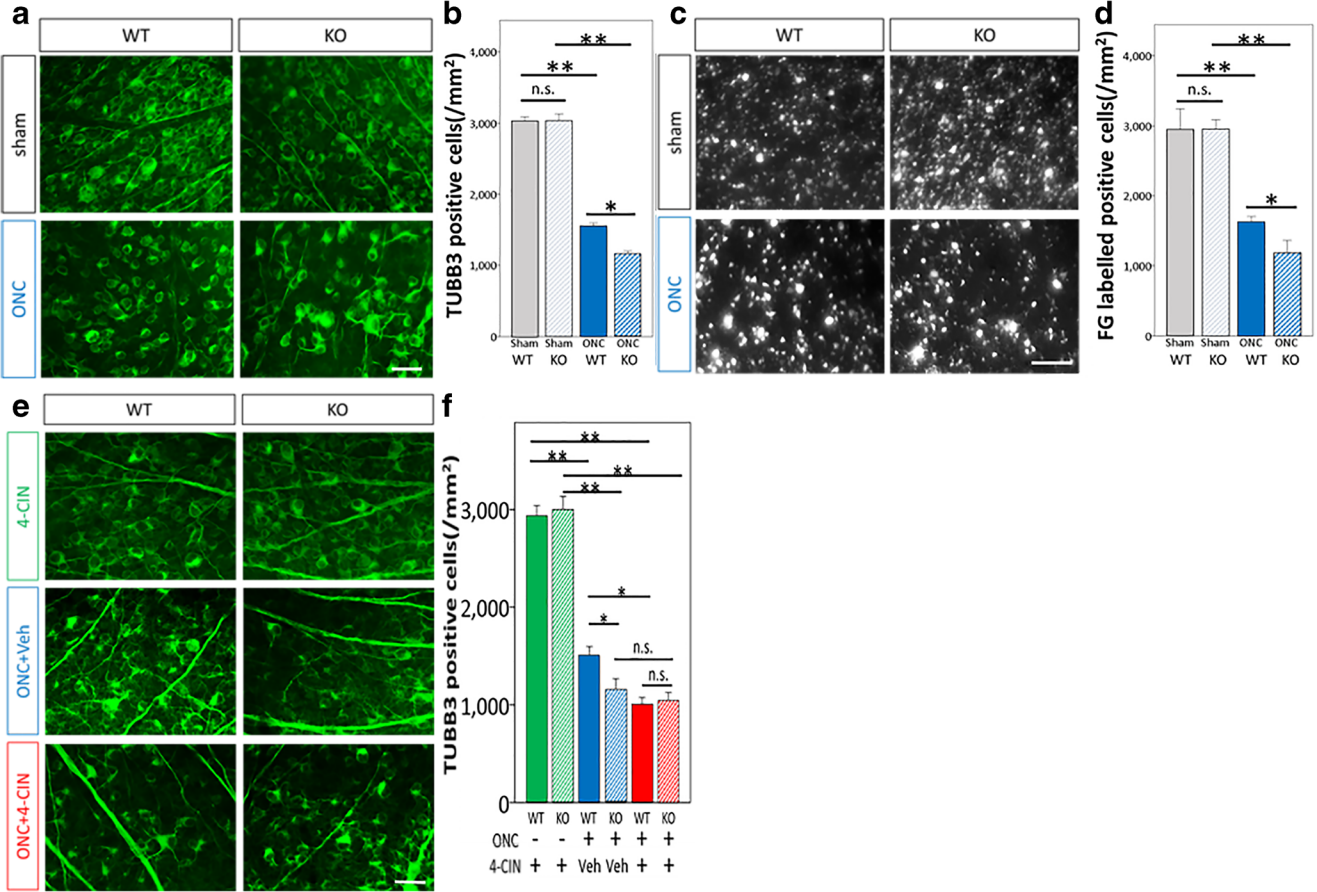
Effects of *Aqp9* gene deletion, ONC, and intravitreal injection of MCT2 inhibitor on RGC density in WT and KO mice. **a** Photomicrographs of TUBB3-immunolabeled cells in whole-mounted retinas of WT and *Aqp9* KO (KO) mice 7 days after a sham operation (sham) or ONC. The scale bar indicates 25 μm. **b** Quantitative comparisons of the density of TUBB3-immunolabeled cells between WT and *Aqp9* KO mice with or without ONC. Error bars indicate standard error of the mean (SEM). *n* = 8 each. **p < 0.001 and *p < 0.01, ANOVA with post hoc Bonferroni test. n.s., not significant. **c** Photomicrographs of retrograde-labeled cells in whole-mounted retinas of WT and *Aqp9* KO mice 7 days after ONC or sham operation with FG injection into the superior colliculus. The scale bar indicates 100 μm. **d** Quantitative comparisons of the density of FG-labeled cells. Error bars designate SEM. The density significantly declined in the WT retina after ONC (*n* = 8, **p < 0.0001). The reduction was more evident in the *Aqp9* KO mouse retinas *(n* = 8, *p < 0.001). n.s., not significant. **e** Photomicrographs of TUBB3-immunolabeled cells on flat-mounted retinas. WT or *Aqp9* KO mice underwent sham operation with a 1-μL intravitreal injection of 10 mM 4-CIN (4-CIN), ONC with a vehicle injection (ONC + Veh), or ONC with the 4-CIN injection (ONC + 4-CIN), and retinas were dissected 7 days later. The scale bar indicates 25 μm. **f** Quantitative comparisons of TUBB3-immunolabeled cell densities among different mouse conditions. Error bars indicate SEM. *n* = 6; **p < 0.0001, *p < 0.05, ANOVA with post hoc Bonferroni test. Veh, vehicle; n.s., not significant. Note that the 4-CIN injection alone did not impact the TUBB3-immunoreactive cell density either in WT or *Aqp9* KO mice and that an additive effect of 4-CIN in the reduced TUBB3-immunoreactive cell density after ONC was observed in WT mice, but not in *Aqp9* KO mice

To investigate whether or not *Aqp9* gene deletion affects RGC survival, we evaluated the RGC density 7 days after ONC using two different methods: anti-TUBB3 immunostaining and retrograde labeling with FG on flat-mounted retinas. In the retinas with sham operation, there was no significant difference in TUBB3-positive RGC densities between WT (2945.4 ± 334.6 cells/mm^2^) and *Aqp9* KO mice (2953.5 ± 149.9 cells/mm^2^), respectively (*p* = 0.97, ANOVA; Fig. 4a, b). Likewise, there was no significant difference in retrograde FG-labeled RGC density in the retinas with sham operation between WT (3025.1 ± 74.7 cells/mm^2^) and *Aqp9* KO mice (3034.5 ± 109.1 cells/mm^2^) (*p* = 0.89; Fig. 4c, d). These findings imply that the *Aqp9* gene deletion per se does not affect RGC survival under physiological conditions, similar to the lack of effect the deletion has on other developmental, metabolic, and behavioral states, as mentioned above.

In contrast, ONC significantly reduced both TUBB3-positive cells (1623.9 ± 91.2 cells/mm^2^) and FG-labeled RGC densities (1549.0.2 ± 51.8 cells/mm^2^) in the WT mouse retinas compared with those with sham operation (*n* = 8, ANOVA with post hoc Bonferroni test *p* < 0.001; Fig. 4a–d), which is in agreement with previous studies [40, 41].

Importantly, the reduction was further increased in *Aqp9* KO mice. In other words, both TUBB3-positive cells (1178.9 ± 204.7 cells/mm^2^) and FG-labeled RGC densities (1148.2 ± 59.0 cells/mm^2^) in *Aqp9* KO mice with ONC were significantly lower than those in WT mice with ONC (*n* = 8, *p* < 0.001; Fig. 4a–d). These results indicate that *Aqp9* gene deletion enhanced RGC death following ONC.

### Intravitreal Injection of an MCT2 Inhibitor Accelerates RGC Death Induced by ONC in WT Mice but Not in *Aqp9* KO Mice

To investigate the effects of double inhibition of AQP9 and MCT2 on RGC survival, an MCT2 inhibitor, 4-CIN, was intravitreally injected in WT and *Aqp9* KO mice with and without ONC, and flat-mounted retinas were immunostained against TUBB3. Without ONC, there were no differences in the densities of TUBB3-positive cells between WT (2932.1 ± 121.2 cells/mm^2^) and *Aqp9* KO mice (2993.9 ± 157.1 cells/mm^2^) that received the 4-CIN intravitreal injection (*n* = 6, *p* = 0.53; ANOVA with the Bonferroni post hoc test; Fig. 4e, f). These values were also comparable to those in mice without 4-CIN injection, as shown above (Fig. 4a–d), indicating the double inhibition of MCT2 and AQP9 does not affect RGC survival under physiological conditions. ONC significantly reduced TUBB3-positive RGC density at 7 days after treatment in WT mice (1496.2 ± 113.6 cells/mm^2^) and, to a greater extent, in *Aqp9* KO mice (1132.8 ± 151.4 cells/mm^2^) with vehicle injection (*n* = 6, *p* < 0.001), which corroborates the aforementioned observations (Fig. 4a–d). Interestingly, when receiving both ONC and 4-CIN injection, the WT mice showed significantly more reduction of TUBB3-positive cell densities (1001.4 ± 85.0 cells/mm^2^) compared with those with ONC and vehicle injection (*n* = 6, *p* < 0.05), whereas *Aqp9* KO mice exhibited a similar density (1039.3 ± 99.5 cells/mm^2^) to those with ONC and vehicle injection (*n* = 6, *p* = 0.53) (Fig. 4e, f). Therefore, the additional inhibition of lactate transport via MCT2 accelerated RGC death in WT mice with ONC that already partially reduced expression of AQP9, the putative another lactate transporter. However, there was no such additive effect of MCT2 inhibition on RGC death induced by ONC in *Aqp9* KO mice, in which the AQP9 that interacts with MCT2 was already almost completely extinguished. This suggests that MCT2 can effectively transport lactate as an energy substrate for RGCs when interacting with AQP9.

### *Aqp9* Gene Deletion Enhances the Reduction of Positive STR Induced by ONC

**Fig. 5:**
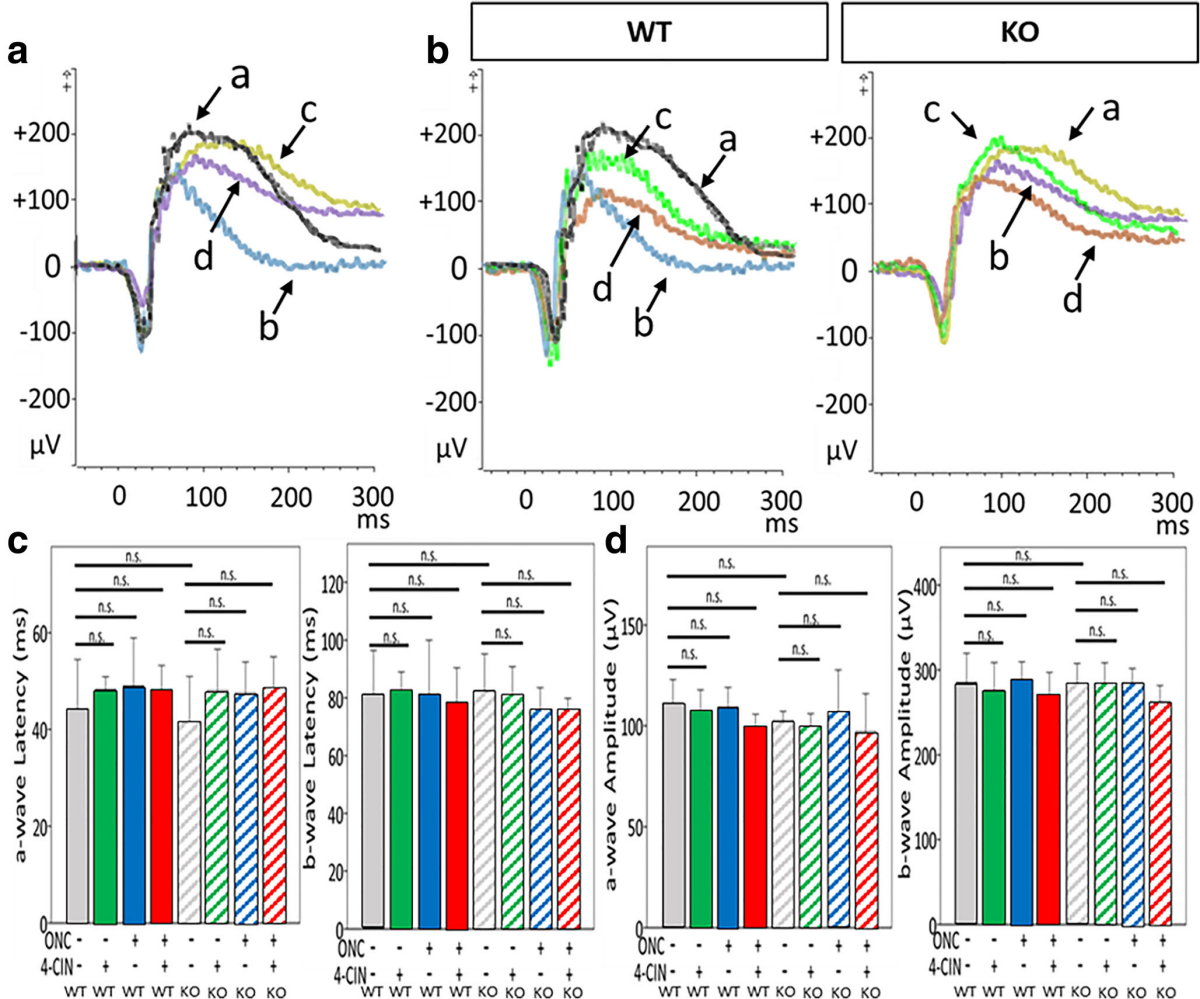
Dark-adapted ERG responses elicited at a light intensity of − 0.15 log sc td s **a** Representative superimposed ERG responses that were recorded from a WT mouse with sham operation (a), a WT mouse with ONC 7 days prior (b), an *Aqp9* KO mouse with sham operation (c), and an *Aqp9* KO mouse after ONC (d). Note similar a- and b-waves of ERGs among the four groups of mice. **b** Representative ERG responses superimposed that were recorded from a WT mouse and an *Aqp9* KO mouse with four different treatments, i.e., sham operation (a), ONC (b), sham operation and intravitreal 4-CIN injection (c), and ONC and 4-CIN injection (d). Note again that there are no apparent differences in a- and b-wave latencies and amplitudes among the treatments in both WT and *Aqp9* KO mice. **c** Quantitative comparisons of latencies of a- and b-waves among groups. **d** Quantitative comparisons of amplitudes of a- and b-waves among groups. Error bars indicate standard error of the mean. n.s., not significant (*n* = 6, ANOVA)

As shown in Fig. 5, dark-adapted ERG with a relatively high intensity stimulation (− 0.15 log sc td s) demonstrated essentially similar amplitudes of a- and b-waves in WT and *Aqp9* KO mice with and without ONC, and with and without 4-CIN, indicating no functional abnormality of photoreceptor and second-order neurons among these mouse groups (*n* = 6, *p* > 0.17 for a-wave amplitude, *p* > 0.10 for b-wave amplitude, ANOVA).

In order to test whether or not *Aqp9* gene deletion has a functional impact on ONC-induced retinal damage, we measured changes in the pSTR amplitudes obtained from a serial intensity series of stimuli, because pSTR is thought to primarily originate from RGC activity [42].

**Fig. 6:**
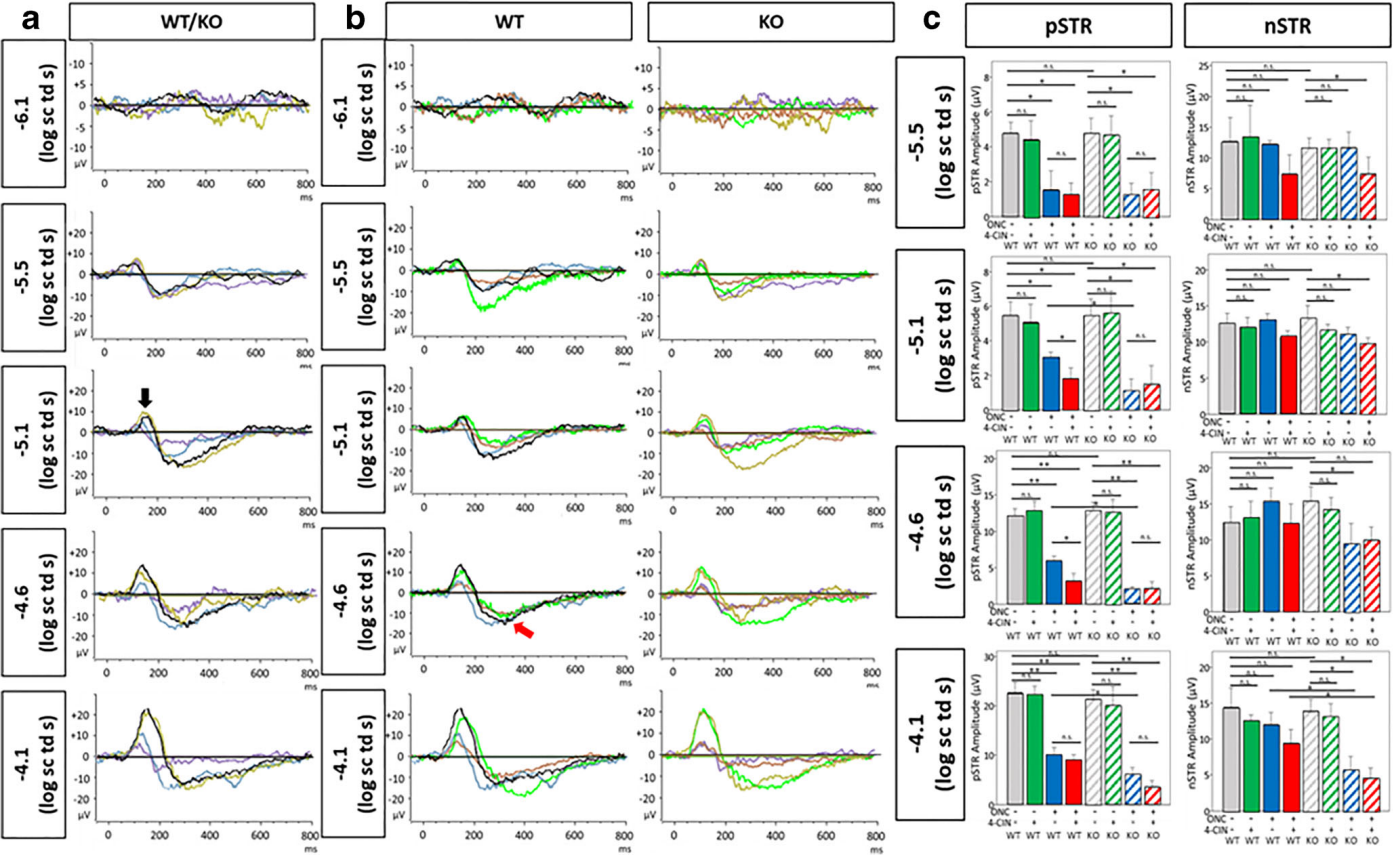
Scotopic threshold responses (STRs) elicited by a series of incremental dim light stimuli. **a** Representative series of superimposed STRs that were recorded from a WT mouse with sham operation (black waves), a WT mouse with ONC (blue waves), an *Aqp9* KO (KO) mouse with sham operation (gold waves), and an *Aqp9* KO mouse after ONC (purple waves). Note that amplitudes of positive STRs (black arrow) are unequivocally reduced in both WT and *Aqp9* KO mice with ONC compared to those with sham operation at light intensities of − 5.1, − 4.6, and − 4.1 log sc td s. **b** Representative series of superimposed STRs that were recorded from a WT mouse and an *Aqp9* KO mouse with four different treatments: sham operation (black waves for the WT mouse and gold waves for the *Aqp9* KO mouse), ONC (blue waves for the WT mouse and purple waves for the *Aqp9* KO mouse), intravitreal 4-CIN injection (green waves for both WT and *Aqp9* KO mice), and ONC and 4-CIN injection (red waves for both mice). The positive STRs are more reduced for the WT mouse with ONC and 4-CIN injection compared with the WT mouse with ONC alone, while the negative STRs (red arrow) are similar among the WT mice with the four different treatments. In contrast, the reduction of the positive STRs in the *Aqp9* KO mice with ONC and 4-CIN injection is similar to that in the *Aqp9* KO mice with ONC alone, while the negative STRs are reduced in the *Aqp9* KO mice with ONC alone and those with ONC and 4-CIN injection at the light intensities of − 5.1, − 4.6, and − 4.1 log sc td s. **c** Quantitative comparisons of amplitudes of pSTR and nSTR among groups. Error bars indicate standard error of the mean. *n* = 6; **p < 0.01, *p < 0.05, ANOVA with post hoc Bonferroni test. n.s., not significant

Figure 6 depicts a scotopic ERG response series elicited by dim light stimuli with incrementally increasing intensities, clearly showing a pSTR recorded at around 110 ms after stimulation, followed by a negative STR (nSTR) at around 200 ms in WT mice that did not undergo ONC or 4-CIN injection. Without ONC, *Aqp9* KO mice exhibited similar pSTR amplitudes during the same intensity series. On the other hand, both WT and *Aqp9* KO mice that underwent ONC exhibited reduced pSTR amplitude (*n* = 6, *p* < 0.01; ANOVA with the Bonferroni test) [29, 42], with the *Aqp9* KO mice showing more reduced amplitude (*p* < 0.05) at the light intensities of − 5.1, − 4.6, and − 4.1 log sc td s. When treated with both ONC and 4-CIN injection, the pSTR amplitude in the WT mice was further reduced compared with ONC alone (*p* < 0.05), while such an additive effect of 4-CIN injection on the reduced pSTR amplitude induced by ONC was not observed in the *Aqp9* KO mice. These pSTR alterations corresponded well to the aforementioned RGC density reduction induced by ONC and 4-CIN injection.

In addition, the nSTR was also reduced in the *Aqp9* KO mice that underwent ONC, particularly with the 4-CIN injection, compared to the WT counterparts at − 4.6 and − 4.1 log sc td s, which may suggest the potential dysfunction of amacrine cells in the *Aqp9* KO mice with ONC alone (*n* = 6, *p* < 0.05) and with ONC and 4-CIN injection (*p* < 0.01).

### *Aqp9* Gene Deletion and ONC Upregulate Expression Levels of GLUT1 and GLUT3

The observations that *Aqp9* gene deletion (and presumably reduced monocarboxylate transport) did not affect the RGC survival under physiological conditions, and that ONC did not result in complete loss of RGCs, imply that metabolism of glucose, the major energy substrate from a classical perspective, could be enhanced as a compensatory mechanism. To test this hypothesis, we first examined retinal GLUT1 and GLUT3 expression levels by immunohistochemistry and immunoblotting. Among the GLUT subtypes, GLUT3, a high-affinity glucose transporter, is reported to be specifically expressed in neurons, while GLUT1 is expresses in astrocytes and endothelial cells [24–26]. Previous studies have also shown that in rat retinas, GLUT1 is expressed in the GCL, INL, and ONL [43], and GLUT3 is diffusely expressed in the IPL and OPL. GLUT3 expression has also been reported in cell bodies in the INL and GCL [44].

**Fig. 7:**
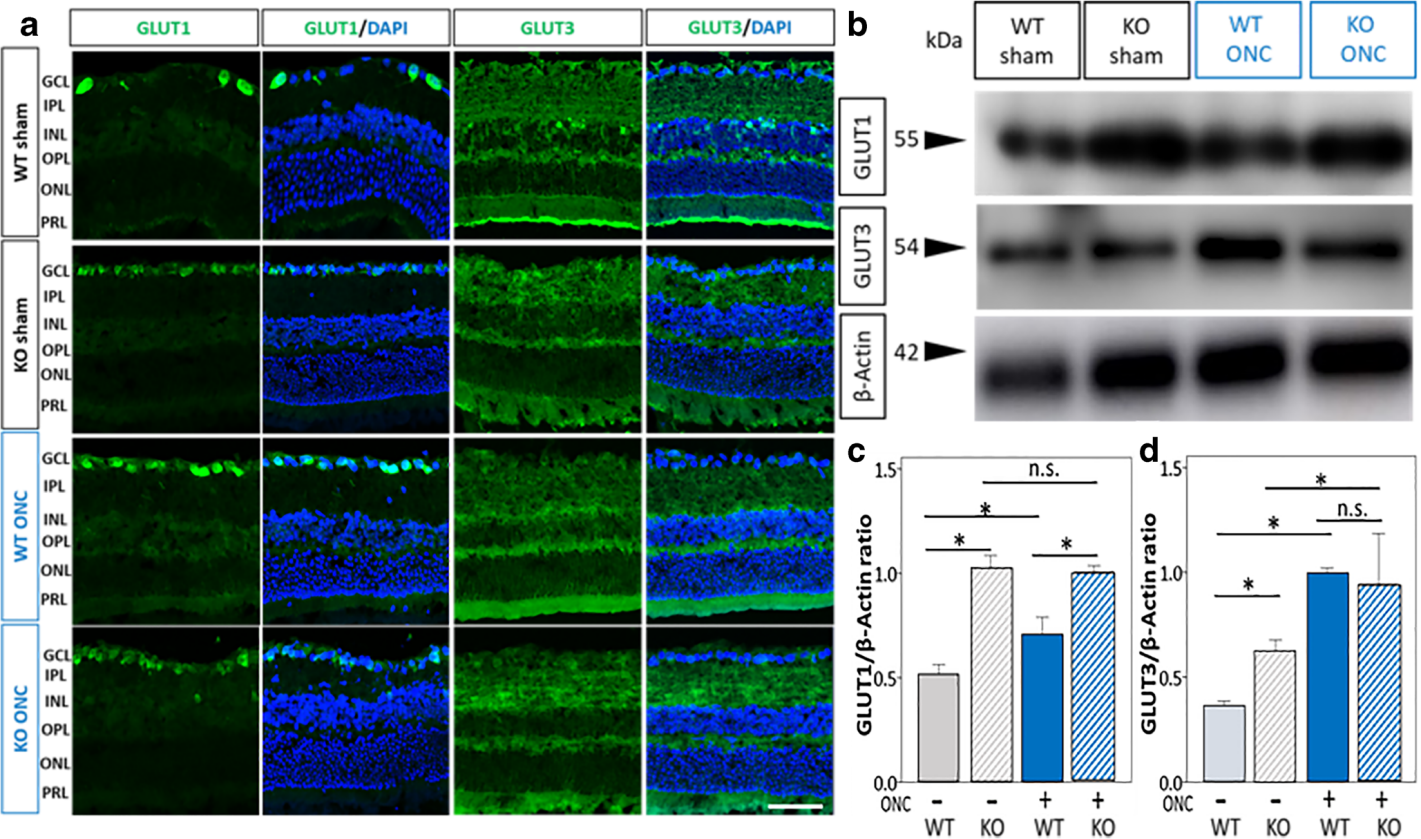
Upregulation of GLUT 1 and GLUT3 expression by *Aqp9* gene deletion and ONC. **a** Immunofluorescence for GLUT1 and GLUT3 in wild-type and *Aqp9* KO mice 7 days after sham operation (WT sham and KO sham, respectively) and ONC (WT ONC and KO ONC, respectively). The scale bar indicates 50 μm. **b** Western blots for GLUT1 and GLUT3 protein expression levels in WT and *Aqp9* KO mice with and without ONC. β-Actin was used as a control. **c**, **d** Quantitative analyses for GLUT1 and GLUT3, respectively, relative to β-actin intensity. Error bars indicate standard error of the mean. *n* = 3; *p < 0.01, ANOVA with post hoc Bonferroni test. n.s., not significant

In our experiment, intense GLUT1 IR was observed in the GCL. Compared to WT mice with sham operation, there was stronger IR in the other three groups of mice, i.e., the WT mice with ONC and the *Aqp9* KO mice with and without ONC (Fig. 7a). There was faint GLUT3 IR at the GCL, IPL, OPL, and PRL in the WT mice with sham operation (Fig. 7a). GLUT3 IR at the OPL and PRL was increased in the *Aqp9* KO mouse retinas with sham operation. In addition, the GLUT3 IR at the IPL was further increased in the WT and *Aqp9* KO mice with ONC compared to the respective counterparts with the sham operation (Fig. 7a). The localization of GLUT1 and GLUT3 found in this study does not contradict the previous reports mentioned above.

Immunoblotting showed that *Aqp9* gene deletion significantly increased GLUT1 expression relative to β-actin, compared with that of WT mice both with and without ONC (*n* = 3, *p* < 0.01, ANOVA with the Bonferroni post hoc test; Fig. 7b, c). The amounts of GLUT1 in the WT mice with ONC were significantly higher than those in the WT mice with sham operation (*n* = 3, *p* = 0.01; Fig. 7b, c), whereas in the *Aqp9* KO mice, there were no significant differences in GLUT1 expression between ONC and sham operation (n = 3, *p* = 0.36; Fig. 7b, c). Relative levels of GLUT3 in the WT mice with ONC were significantly higher than the WT mice with sham operation (*n* = 3, *p* < 0.01; Fig. 7b, d). *Aqp9* gene deletion per se significantly increased the GLUT3 levels compared with the WT mice without ONC (*n* = 3, *p* < 0.001). ONC further increased GLUT3 levels in the *Aqp9* KO mice (*n* = 3, *p* = 0.02; Fig. 7b, d). However, there was no significant differences in the GLUT3 expression levels between the WT mice with ONC and the *Aqp9* KO mice with ONC (*n* = 3, *p* = 0.41; Fig. 7b, d).

ONC upregulated GLUT1 and GLUT3 expression levels, both in the WT and *Aqp9* KO mice (Fig. 5b–d). However, the magnitude of upregulation was different between GLUT1 and GLUT3 and among the mouse conditions. In other words, the magnitude of increase in GLUT1 expression induced by *Aqp9* gene deletion was greater than that induced by ONC, whereas the magnitude of increase in GLUT3 expression induced by ONC was greater than that induced by *Aqp9* gene deletion.

Collectively, *Aqp9* gene deletion predominantly increased the GLUT1 expression particularly in the GCL, while ONC predominantly increased GLUT3 expression particularly in the IPL, OPL, and PRL.

### *Aqp9* Gene Deletion Lowers l-Lactate Concentrations and Increases d-Glucose Concentrations in the Retina

**Fig. 8:**
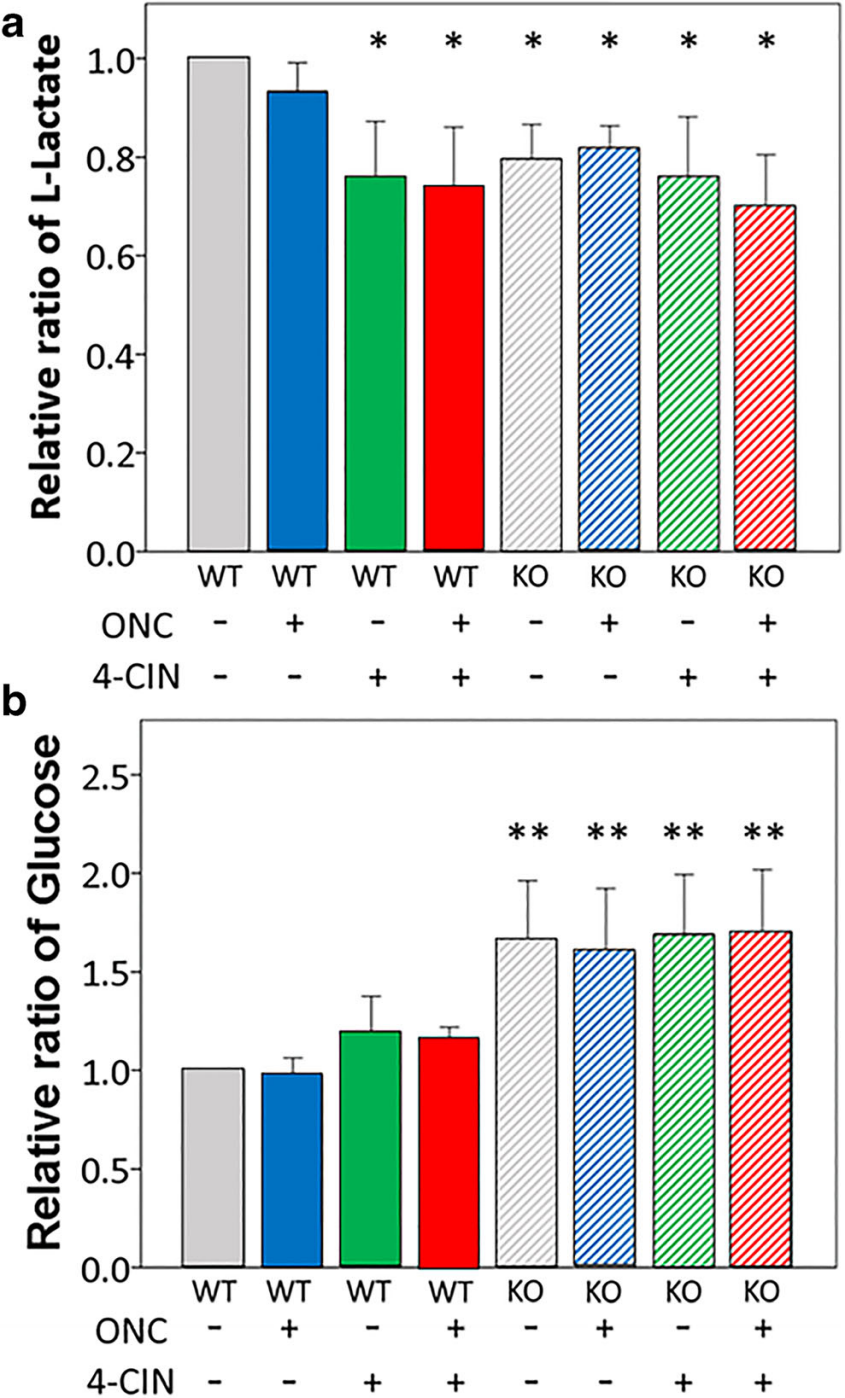
Relative ratios of l-lactate and d-glucose concentrations in mouse retinas measured by colorimetric assays. **a** Retinal l-lactate concentrations in mice with different conditions relative to those in WT mice that underwent sham operation with an intravitreal injection of vehicle (control). **b** Retinal d-glucose concentrations in mice with different conditions relative to d-glucose concentrations in controls. WT, wild-type mice; KO, *Aqp9* knockout mice; ONC, optic nerve crush performed 7 days prior; 4-CIN, α-cyano-4-hydroxycinnamate. Error bars indicate standard error of the mean. *n* = 8; **p < 0.01, *p < 0.05, ANOVA with post hoc Bonferroni test. n.s., not significant

To evaluate whether or not alterations of monocarboxylate and glucose transporters affect substrate utilization in the retina, we measured the l-lactate and d-glucose concentrations relative to a control condition (WT mice with sham operation) by colorimetric assays (Fig. 8).

Compared with controls, neither l-lactate nor d-glucose concentrations changed in the WT mice with ONC. Intravitreal injection of 4-CIN and *Aqp9* gene deletion significantly reduced the relative concentration of l-lactate in the retina. However, there was no additive effect of MCT2 inhibition and *Aqp9* gene deletion on the l-lactate concentration, as the l-lactate concentrations were not different between *Aqp9* KO mice without and with 4-CIN injection (*n* = 8, *p* = 0.63, ANOVA). ONC did not have an additional effect on the l-lactate concentrations in the other mouse conditions.

In a clear contrast, intravitreal injection of 4-CIN or the combination of ONC and 4-CIN injection in WT mice did not impact d-glucose concentrations relative to controls, whereas *Aqp9* gene deletion significantly increased the relative levels of d-glucose by approximately 1.5-fold (*n* = 8, *p* < 0.001 ANOVA with the Bonferroni post hoc test). This increase was not additionally modified by ONC or 4-CIN injection (*n* = 8, *p* = 0.70 or *p* = 0.60, respectively).

Taken together, inhibition of MCT2 or AQP9 reduced the intraretinal l-lactate concentration, regardless of whether the mice received ONC, while *Aqp9* deletion, but not MCT2 inhibition, increased intraretinal d-glucose concentration, regardless of whether mice received ONC. The reduced intraretinal lactate concentrations in mice with the 4-CIN injection or *Aqp9* gene deletion may be due to the pharmacologically or genetically downregulated lactate transporter expression in the retina, whereas the increased intraretinal glucose concentrations may correspond to the upregulated GLUT1 and GLUT3 expression in *Aqp9* KO mouse retinas mentioned above.

## Discussion

Results confirm that RGCs and retinal astrocytes express AQP9 and show that ONC reduces the expression of AQP9, with a greater reduction found in the astrocytes of WT mice. When carefully reviewing our previous studies, we noticed that not only RGC cell bodies but also superficial layers above them, expressed AQP9 in control rat retinas, and that both elevated IOP and optic nerve transection reduced AQP9 expression in RGCs together with almost completely extinguished AQP9 expression in the superficial layers (see Figure 10B in [11] and Figure 8A in [12]. Given that the retina is a part of the central nervous system and that astrocytes in the brain are reported to express AQP9 [5–9], it is not surprising to detect astrocytic expression of AQP9 in the retina. Tran et al. [13] reported that human glaucomatous eyes also had reduced AQP9 expression in RGCs, indicating that reduced expression of AQP9 induced by stress on the optic nerve may be common phenomenon across species.

The higher rate of ONC-induced RGC death in *Aqp9* KO mice compared to WT mice suggests a dose dependency of AQP9 expression on RGC survival and functional maintenance. It is noteworthy that functional alterations evaluated by pSTR support such histological observations. Further, the fact that the increase in RGC death associated with downregulated AQP9 expression correlated with a reduction of intraretinal lactate concentrations and an increase in intraretinal glucose concentrations to some degree indicates that AQP9 plays a critical role in lactate transport as an energy substrate for RGCs and that downregulation of AQP9 expression induced by ONC impairs lactate transport, resulting in increased RGC death. These lines of evidence further support the notion that ANLS between astrocytes and RGCs in the inner neural retina plays a role in RGC survival, as our previous in vivo [11, 12] and in vitro [12, 14] data have demonstrated.

Notwithstanding, *Aqp9* gene deletion per se did not lead to RGC death and dysfunction detectable by ERG, indicating that additional routes for lactate transport exist that are indispensable for survival and normal function of RGCs. This study has in fact demonstrated that all of the three isoforms of MCTs (MCT1, MCT2, and MCT4) were expressed in GCL, in addition to the retinal layers, where they were reported to be expressed in previous studies [20–22]. Given the reported distribution and presumed function of MCTs in the brain, where astrocytes export lactate mainly via MCTs 1 and 4, while neurons imports lactate predominantly through MCT2 [17, 18], it is not surprising that MCT2 was expressed in the GCL, in which the third-order RGC neurons reside. Variability in the IR patterns of MCTs in the GCL among studies might be due to differences in epitope recognition of the antibodies and animal species used.

**Fig. 9:**
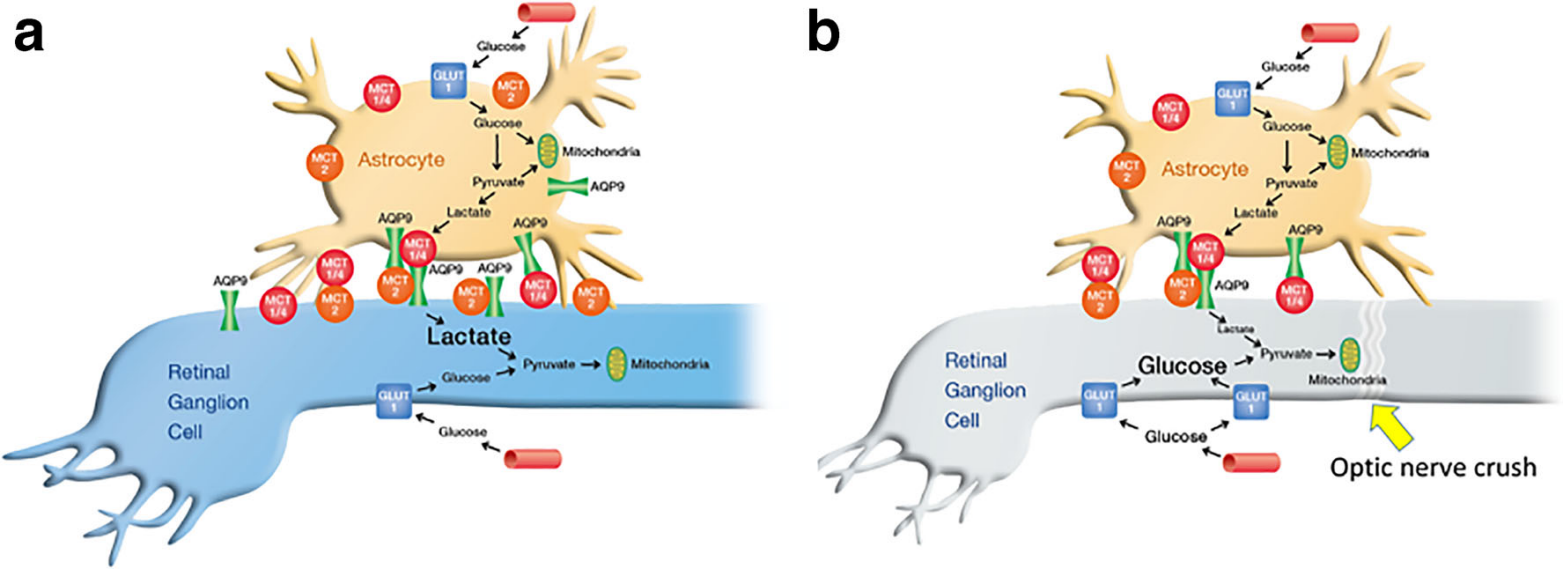
The energy transportation in normal (**a**) and optic nerve crush (ONC) (**b**) between astrocytes and retinal ganglion cells (RGCs). Aquaporin 9 (AQP9) and monocarboxylate transporters (MCTs) along with lactate transport from astrocyte to RGCs. ONC lowers the expression of AQP9 and all isoforms of MCT and disrupts interactions of AQP9 with MCTs1 and 4, which lead to the lower concentration of lactate in the retinal tissue. To compensate for reduced energy supply via lactate, glucose transporter (GLUT) expression is upregulated to increase the direct uptake of glucose into RGCs

Figure 9 shows a schematic diagram to illustrate the relationship between AQP9 and MCTs and between astrocytes and RGCs in a healthy state and following ONC. One of the most important observations in this study is that MCT1, MCT2, and MCT4 at least partly interacted with AQP9 in the retina. Evidence is accumulating that AQP9 functions through protein-protein interactions [45]. Previously, MCT1 was reported to require the accessory protein CD147, or basigin, for trafficking to the plasma membrane to become a hetereomeric transporter [46]. Mice lacking CD147 exhibit severely reduced ERG responses, progressive photoreceptor degeneration, and reduced levels of MCTs1, 3, and 4 in the retina [20]. This study suggests that MCTs drive ANLS not only with CD147, but also in concert with AQP9, at least partly, in the physiological conditions of the retina. Further study is necessary to elucidate the interplay among CD147, MCTs, and AQP9.

In the meantime, the findings that total concentrations of MCTs were either unchanged (MCT1) or upregulated (MCT2 and MCT4) in *Aqp9* KO mice indicate that not all MCT proteins couple with AQP9, but a fraction of MCTs is expressed independently from AQP9 in a subset of sites in cells and/or organelles. GLUT 1 and GLUT3 expression levels were upregulated, which accompanied elevated intraretinal concentrations of glucose, in the *Aqp9* KO mice. These findings suggest that survival and function of the inner retina are maintained under physiological conditions in the absence of AQP9 either by a compensatory increase in glucose utility or in lactate transport through routes that do not involve AQP9 such as via MCTs, or both. This supports the idea put forth by Erlichman et al. that neurons may metabolize a combination of glucose and lactate, or other substrates such as pyruvate and ketone bodies, depending on the availability of each of these particular substrates [30].

In this study, ONC dramatically reduced the amounts of MCT1 and MCT4 that were co-immunoprecipitated with AQP9, whereas it did not affect the amount of MCT2 that was co-immunoprecipitated with AQP9 in WT mice. These findings corroborate the immunolabeling data showing that AQP9 IR co-localized in RBPMS-positive cells was relatively well preserved, but that co-localization in GFAP-positive cells was substantially reduced in WT mice with ONC, because MCT1 and MCT4 are mainly astrocyte-specific isoforms, whereas MCT2 is the main neuronal isoform. In our study, we validated that MCT2 co-expresses with RGCs by co-immunostaining methods with MCT2 and RGC markers (Brn3a) in Appendix Fig. 10. The maintained expression of MCT2 that probably interacted with preserved AQP9 in RGCs may have contributed to protect RGCs from death induced by ONC in the WT mice, because these RGCs were capable of taking up circulating lactate in blood rather than lactate locally released from astrocytes [2, 17, 47] which was presumably reduced due to the downregulated MCT1 and MCT4 expression. Upregulation of total MCT2 levels observed in the *Aqp9* KO mice with sham operation may also reflect a compensatory response to presumably reduced expression of astrocytic MCT1 and MCT 4 that coincided with reduced AQP9 expression and subsequent decrease in local lactate release from astrocytes. These observations may link to the previous report by Tescarollo et al. [48] showing that upregulated expression levels of AQP9 and MCT2 in hippocampal neurons after glutamate exposure coincided with reduced glucose metabolism. In fact, recently Mohammad Harun-Or-Rashid et al. have conducted a study wherein the overexpression of MCT2 demonstrated the neuroprotective effect in the retina and optic nerve [49].

The fact that the injection of 4-CIN, the MCT2 inhibitor, increased RGC death and reduced pSTR amplitude induced by ONC in WT mice further supports the idea that multiple lactate transport pathways may act complimentarily with each other to promote RGC survival and maintain their function. Further, because ONC alone led to maximal RGC death and pSTR amplitude reduction with no additive effect of 4-CIN injection in the *Aqp9* KO mouse retina, which had no AQP9 to interact with MCT2 but enhanced total MCT2 expression, MCT2 may more efficiently import lactate when it interacts with AQP9 under stress.

Conversely, as AQP9 and MCT2 appear to synergistically function to some degree, exogenous application of lactate may potentially enhance survival and function of RGCs. In fact, evidence is accumulating that such an application ameliorated metabolic deficiencies in traumatized brain [50, 51]. Given the lack of effective treatments for traumatic optic neuropathy, the exogenous application of lactate might be a future target of study for the treatment of patients with traumatic optic neuropathy.

Harum-Or-Rashid et al. [26] reported that the rodent optic nerve in a chronic glaucoma model exhibited reduced expression of GLUT1, MCT1, MCT2, and MCT4 together with lactate concentrations and that an acute elevated IOP model also showed reduced expression of MCT1 and MCT2. The present observations of downregulation of MCT1 and MCT2 in the mouse retina after ONC is in agreement with their reports, while upregulated expression of GLUT1 and GLUT3 in the present study contradicts their data. The discrepancy between these studies regarding the responses observed for GLUT expression may derive from differences in the types and severity of stress inflicted on the optic nerve and/or in the samples analyzed (i.e., optic nerve vs. retina).

An additional finding of interest in the present study is that *Aqp9* KO mice that underwent both ONC and 4-CIN injection exhibited significant reductions of nSTR amplitude. It is presumed that nSTR originates from amacrine cells [37, 41]. As mentioned above, it was catecholaminergic amacrine cells that were originally found to express AQP9 in the retina [10]. Because it is plausible that *Aqp9* KO mice have lost AQP9 in amacrine cells in addition to RGCs and astrocytes, double stresses of ONC and MCT2 inhibition by 4-CIN injection may have led to dysfunction of amacrine cells in these mice.

The current study has two major limitations. First is the possible incompleteness of *Aqp9* gene deletion. In Fig. 2, mRNA and proteins for AQP9 were detectable, though faintly, in the *Aqp9* KO mice. The previous studies [9, 27] also indicated that there was faint but positive labeling of antigen and mRNA of AQP9 in the same *Aqp9* KO mice with the use of immunostaining and in situ hybridization in several tissues such as the brain as well as the liver, epididymis, testis, skin, spleen, muscle, spinal cord, ovaries, and intestine. They speculated that these detections were nonspecific responses. However, AQP9 molecules are deemed to contain various isoforms that are expressed in the mitochondria and cell membrane, which may be also different in tissue or cell types. Thus, it is not surprising that in the current *Aqp9* KO mice, expressions of all types of AQP9 isoforms may not be totally extinguished. Nonetheless, the AQP9 function was minimal, if any, in the *Aqp9* KO mice given the very faint expression. Another limitation is a lack of direct evidence of affinity strength of MCTs with AQP9. Although immunoprecipitation data clearly show the mobile interaction of AQP9 and MCTs, ideally, a biochemical assay should be performed to validate this issue.

In conclusion, *Aqp9* gene deletion does not affect survival or function of RGCs under physiological conditions, but does increase RGC death and dysfunction under stress. AQP9 interacts at least partly with MCTs, presumably to transport lactate as an energy substrate. These lines of evidence suggest that AQP9 may play a role in ANLS in the inner retina in order to promote survival and maintain function of RGCs.

## Figures and Tables

**Fig. 10:**
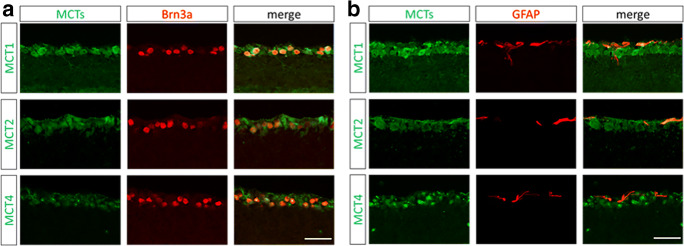
Expression of Brn3a/GFAP and monocarboxylate transporters (MCT)1, 2, and 4 in ganglion cell layer (GCL). **a** MCTs and Brn3a immunoreactivity (IR) in inner retinas. **b** MCTs and GFAP immunoreactivity (IR) in inner retinas. Scale bars indicate 50 μm

## Data Availability

The datasets used and/or analyzed during the current study are available from the corresponding author on reasonable request.

## Abbreviations

ANLS: Astrocyte-to-neuron lactate shuttle
ANOVA: One- or two-way analysis of variance
AQP9: Aquaporin 9
DAPI: 4′,6-Diamidino-2-phenylindole
ERG: Electroretinogram
FG: FluoroGold
GCL: Ganglion cell layer
Gapdh: Glyceraldehyde 3-phosphate dehydrogenase
GFAP: Glial fibrillary acidic protein
GLUT: Glucose transporter
INL: Inner nuclear layer
IOP: Intraocular pressure
IPL: Inner plexiform layers
IR: Immunoreactivity
KO: Knockout
MCT: Monocarboxylate transporters
nSTR: Negative scotopic threshold response
ONC: Optic nerve crush
ONL: Outer nuclear layer
OPL: Outer plexiform layer
PRL: Photoreceptor layer
pSTR: Positive scotopic threshold response
RBPMS: RNA-binding protein with multiple splicing
RGC: Retinal ganglion cell
RPE: Retinal pigment epithelium
RT-PCR: Reverse transcription polymerase chain reaction
STR: Scotopic threshold response
TUBB3: Anti-tubulin β3
WT: Wild type
4-CIN: α-Cyano-4-hydroxycinnamate
